# Supplementary material for: Gaussian process inference modelling of dynamic robot control for expressive piano playing
Source: PLoS One. 2020 Aug 14;15(8):e0237826. doi: 10.1371/journal.pone.0237826 (PMC7428139; doi:10.1371/journal.pone.0237826)
Supplement: Attachment — Submitted filename: reply to reviewers.pdf [file pone.0237826.s001.pdf]

Reply to reviewers

PONE-D-20-12759

Gaussian Process inference modelling of dynamic robot control for expressive piano playing

We wish to thank the reviewer for the insightful comments. We believe that by addressing the key points mentioned by the reviewer, the quality of the paper has since improved. Thanks to the reviewer's comments it is now clearer the context in which the experiments were done, including how the 'human' playing was investigated, and how the experiments were carried out. The changes are highlighted in red in the revised text "manuscript_tracked.pdf". The submission folder has also been revised accordingly, with high resolutions figures.

Reviewer #1: Playing piano is a complex skill, and make robot own this skill is a hot topic in recent years. This paper develop the gaussian process inference modelling of dynamic robot control for expressive piano playing. The work is interesting and the experiments are sufficient to prove the proposed methods. However I have some questions,

(1) the paper "investigates the complex dynamics of the embodied interactions between a human and piano, in order to gain insights into the nature of humans' physical dexterity and adaptability". can you explain how do you investigate? with some date glove like Ref."3D human gesture capturing and recognition by the IMMU-based data glove, Neurocomputing, 2018, 277：198-207." or visual way like " Vision-based teleoperation of shadow dexterous hand using end-to-end deep neural network, International Conference on Robotics and Automation (ICRA), 2019, 416-422.", please help give the explanation.

We wish to thank the reviewer for this comment. The paper was indeed not very clear as to how the relationship between the human piano playing and the robot playing was captured. The human playing, as well as the robot playing, were not captured by any tracking system. In terms of piano playing, the work focused instead on the outcome of a certain *key-press* action, rather than how this was performed by the human. In this context, the human hand was not tracked. Instead, the *result* of the human key-press action was captured by the piano itself in terms of key-press velocity, key-press hold time, key-press release velocity, and key-press wait time. These parameters were initially generated by the MuseScore software, and later attempted by both the human and the robot for comparison.
Human hand tracking technology is indeed a very exciting future direction for this research, one which is ongoing at the moment, and the authors agree it should be mentioned in the manuscript.

The manuscript has been revised following the reviewer's comment. We have clarified the explanation of the human and robot playing style capture in Section 3.3, paragraph 1, clarifying no human hand tracking was used for the experiments. The paragraph now reads:

"Finally, we investigate the ability of the robot to perform the 10 different playing styles in Fig.6a as compared to an expert human pianist. We use the controllers optimised by the GP-based framework developed. The human performer is a veteran pianist with 15 years of history in piano playing. To perform this comparison, we do not track the human hand trajectory whilst performing the key-press. Instead, we focus directly on the outcome of this trajectory by recording MIDI events during the human playing, and directly comparing these events with the ones triggered by the robot playing.

Upon listening to the sound output the pianist is made to reproduce the note on the piano. We collect 10 different key-press samples at 40 beats per minute (BPM) or 1 key-press every 1.5s, performed 4 times by the human pianist, for each playing style, so (...)"

We have also augmented the Discussion and Conclusion (Section 4), paragraph 4, mentioning human hand tracking within the context of piano playing as a future direction for this project, we have included the suggested references and expanded on the benefits of this future direction. The section now reads:

"Finally we compare the ability of the robot to approximate each of the playing styles, with respect to an expert human player. We show the comparison sheds some light to several interesting aspects of robotic piano playing. The robot is capable of performing comparatively to the human player in the syles of normal, tenuto, staccato and pppp, largely due to the precise control at low speeds, and clock waiting times. The human-player, however, exhibits a much more dynamic and varied playing, which allows them to achieve lower style error to the MuseScore generated playing styles in ff, f, mp, and p. These styles, in fact, require higher downward key-press speeds and dynamic playing. A limitation factor, in this context, is the nature of the comparison, which was applied directly to the MIDI outcome of a key-press. A future interesting direction involves the employment of technology capable to track the trajectory of motion of the human hand whilst performing a key-press[21, 22]. This trajectory can then be compared to the optimized key-press performed by the robot to gain additional insights as to how machine can approach human capabilities for instrument playing."

(2) As shown in Fig.3, the movement of x-axis is before the movement of z, why are they moving simultaneously?

We wish to thank the reviewer for this comment. From Fig.3 it is not very clear how the movement across the x and z axis unfolds through the course of the experiments. In the context of the experiments, the movement on the x and z axis do not happen simultaneously. The motion on the x axis is the first to happen, where the robot alights its end-effector with the piano key to press. Afterwards, repetitive experiments can be triggered, where the z and Rx axis can be controlled for each key-press experiment. For each experiment, the Rx axis is the first to be controlled, where the finger will assume a specific rotation position before performing the key-press. One this rotation position is reached, the robot can move along the z axis to perform the key press, before returning to the starting position, and repeating the experiments. We have revised and improved the explanation in Section 2.3, where Paragraph 1 now reads:

"The robot was controlled in Cartesian coordinates at 125Hz, acting upon the Z and Rx tool axis, to generate the desired contact between the end-effector and piano key for a key-press. The x axis and Rx axis were controlled first, to align the robot's end-effector with the key to be pressed (x), and assume a rotation about the fingertip (Rx). Subsequently the robot was controlled via the z axis, to perform a key-press. A hybrid sinusoidal displacement profile was generated for the z axis, parameterized in both amplitude and frequency. The alignment, controlled by the x-axis, does not generally influence a key-press. The Rx and z axis, instead, influence each key-press uniquely. A total of 5 parameters were thus used to control these two axes during each key-press experiment (d_v=5), i.e. Rx, f_1, f_2, t_1, t_2 (Fig.3b)."

While Paragraph 3 now reads:

"Here, 't' is the time, in seconds, elapsed since the start of the touch experiment and Az defines the amplitude of the generated sinusoidal displacement for the key press, and it is here set to 32mm throughout the experiments. Additionally, a parameter Rx sets the angle of rotation of the end-effector, between 0° and 90° throughout the experiments (Fig3a). The x and Rx axis of motion controls

the robot's ability to shift key along the piano as it plays different notes. The control on the x axis is achieved through (...)"

(3) Please provide the high-quality figures.

Thank you for this comment, we have provided a high quality versions of all the figures together with the revised submission.
